# Nitric oxide induces cell death in canine cruciate ligament cells by activation of tyrosine kinase and reactive oxygen species

**DOI:** 10.1186/1746-6148-8-40

**Published:** 2012-03-29

**Authors:** Simone Forterre, Andreas Zurbriggen, David Spreng

**Affiliations:** 1 Division of Small Animal Surgery and Orthopedics, Vetsuisse Faculty Bern, Department of Clinical Veterinary Medicine, University of Bern, Länggassstrasse 128, 3012, Bern, Switzerland; 2 Division of Clinical Research, Department of Clinical Research and Veterinary Public Health, University of Bern, Bremgartenstrasse 109, 3012, Bern, Switzerland

**Keywords:** Cranial cruciate ligament rupture, Dogs, Apoptosis, Nitric oxide, Sodium nitroprusside, Reactive oxygen species, Tyrosine kinase

## Abstract

**Background:**

There is increasing evidence suggesting that development of progressive canine cranial cruciate ligament (CCL) rupture involves a gradual degeneration of the CCL itself, initiated by a combination of factors, ranging from mechanical to biochemical. To date, knowledge is lacking to what extent cruciate disease results from abnormal biomechanics on a normal ligament or contrary how far preliminary alterations of the ligament due to biochemical factors provoke abnormal biomechanics. This study is focused on nitric oxide (NO), one of the potential biochemical factors. The NO-donor sodium nitroprusside (SNP) has been used to study NO-dependent cell death in canine cranial and caudal cruciate ligament cells and to characterize signaling mechanisms during NO-stimulation.

**Results:**

Sodium nitroprusside increased apoptotic cell death dose- and time-dependently in cruciate ligamentocytes. Cells from the CCL were more susceptible to apoptosis than CaCL cells. Caspase-3 processing in response to SNP was not detected. Testing major upstream and signal transducing pathways, NO-induced cruciate ligament cell death seemed to be mediated on different levels. Specific inhibition of tyrosine kinase significantly decreased SNP-induced cell death. Mitogen activated protein kinase ERK1 and 2 are activated upon NO and provide anti-apoptotic signals whereas p38 kinase and protein kinase C are not involved. Moreover, data showed that the inhibition reactive oxygen species (ROS) significantly reduced the level of cruciate ligament cell death.

**Conclusions:**

Our data support the hypothesis that canine cruciate ligamentocytes, independently from their origin (CCL or CaCL) follow crucial signaling pathways involved in NO-induced cell death. However, the difference on susceptibility upon NO-mediated apoptosis seems to be dependent on other pathways than on these tested in the present study. In both, CCL and CaCL, the activation of the tyrosine kinase and the generation of ROS reveal important signaling pathways. In perspective, new efforts to prevent the development and progression of cruciate disease may include strategies aimed at reducing ROS.

## Background

Cranial cruciate ligament (CCL) rupture is one of the most common orthopedic diseases among dogs and an important cause of osteoarthritis (OA). The majority of dogs rupture their CCL during normal daily activities due to progressive degenerative changes within the ligament itself [1]. Mechanical factors such as straight tibial plateau angle, distal femoral torsion, tibial torsion, as well as intercondylar notch stenosis have been associated with CCL disease [2-4]. There is no doubt that biomechanical factors, among others, are likely to play an important role in the disease process [5] but the true effect on the ethiopathogenesis is currently unknown. There is strong evidence that the degenerative changes observed in CCL disease are due to a combination of factors, ranging from mechanical to biochemical. Developmental, immune-mediated disorders, genetic components, as well as impaired synthesis and turnover of cells and extracellular matrix have been implicated as biological factors [6-8]. To date, knowledge is lacking to what extent cruciate disease results from abnormal biomechanics on a normal ligament or contrary how far preliminary alterations of the ligament due to biochemical factors provoke abnormal biomechanics. A lot of research has focused on stifle joint stabilization as therapeutical intervention but markedly less studies investigated the role of biochemical factors.

Nitric oxide (NO) is one of these biochemical factors considered to be involved in the canine cruciate disease and to influence apoptosis in the CCL [9,10]. Under normal physiological conditions low levels of NO are produced by constitutive nitric oxide synthases (cNOS) to regulate a number of homeostatic processes, whereas the generation of larger quantities of NO by inducible NOS (iNOS) accounts for an inflammatory setting [11]. The role of NO has been investigated excessively in cartilage. Studies demonstrate that NO increases the number of chondrocyte death, which correlates with the extent of cartilage degradation in human OA [12-14] as well as in experimental induced OA in rabbits [15] and in dogs [16,17]. Most studies linking NO and chondrocyte death are based on the use of exogenous NO-donors such as sodium nitroprusside (SNP) [13,15,18,19]. Nitric oxide has been shown to affect crucial intracellular signaling pathways in various human and animal cells of the joint [13,19-21]. In contrast to cartilage tissue, only a few studies investigated the impact of NO on the degeneration of the canine CCL [9,10,22,23], although in the CCL of dogs an excess production of NO has been documented [9]. Furthermore, the intracellular signaling by which NO mediates apoptosis in the CCL remains to be elucidated.

The objective of the presented study was to test the hypothesis that known NO-mediated signaling pathways from chondrocytes or synoviocytes are also activated in canine cruciate ligament cells. In a previous study our group demonstrated a different susceptibility to apoptosis between CCL and caudal cruciate ligament (CaCL) cells [24]. In order to prove if a relationship between susceptibility and signaling exists, we examined specific intracellular signaling pathways involved in NO-induced cell death in canine CCL and CaCL cells. Because there is no recent literature of the NO-pathways in canine cruciate ligamentocytes, specifically ligamentocyte cultures, we decided to compare our results also to publications on human and rabbit joint tissue cultures.

## Methods

### Materials

All reagents for the tissue preparation and assays were purchased from Sigma-Aldrich (Buchs, Switzerland) unless otherwise stated. Sodium nitroprusside (SNP), SB-202190, SN-50, and NS-398 were purchased from Enzo Life Sciences (Lausen, Switzerland). Carboxy-PTIO, taxifolin, genistein (4,5,7-Trihydroxyisoflavone), calphostin C, and uric acid were from Calbiochem (Merck, Switzerland). PD98059 was obtained from Cell Signaling (BioConcept, Allschwil, Switzerland).

### Cell preparation

Tissues from the CCL and CaCL of 8 one day-old beagle dogs (5 male, 3 female) were obtained within 1 h of death in accordance with review board approval by the Animal Care and Experimentation Committee of the Canton of Bern, Switzerland (No 56/06). Ligaments were harvested under aseptic conditions and placed in sterile DMEM medium. A portion of the ligament at both the tibial and femoral ends was trimmed and discarded. The outer synovial layer was removed via sharp dissection and the ligaments were cut into 0.1 – 0.2 mm pieces. For cruciate ligamentocyte isolation, canine CCL and CaCL pieces were digested with collagenase type IV and cultivated in DMEM supplemented with 15% fetal calf serum (FCS), 250 μM ascorbic acid and antibiotics as previously reported [24]. At 80% confluence, cells were harvested after trypsin/EDTA treatment and frozen in 20% FCS and 10% DMSO until use. After thawing and passaging again, cells from the fourth passage were synchronized by 10% FCS-DMEM for 1 day and then used in the following experiments. Cell cultures up to the fifth passage were investigated to confirm purity using specific fibroblast antibodies. Canine cruciate ligamentocytes were identified by immunohistochemistry with antibodies against fibronectin (F3648) and collagen I (6308, Abcam, USA) and compared to the staining in explants of CCL and CaCL of dogs as described before [24]. Human OA chondrocytes (kindly provided by S. Kohl) served as positive controls for prostaglandin (PG) E_2_ measurements.

### Experimental culture conditions

Because of its ability to induce apoptosis in canine cruciate ligamentocytes, SNP was chosen as a pro-apoptotic agent. For the experiments on the SNP dose response, cells were treated with various concentrations of SNP in 10% FCS-DMEM for 18 h. A time course of response was also investigated by incubating cells with SNP for the indicated time period (6–24 h). To explore signaling cascades in SNP-induced cell death, inhibitors of different pathways were used as mentioned below. Therefore, canine cruciate ligamentocytes (40 × 10^3^ cells/96-well for MTT or 10^6^ cells/6-well for flow cytometry and immunoblot) were preincubated with each inhibitor for 2 h, and then SNP concentrations (0–0.5 mM; as indicated) was added directly to the cultures and allowed to incubate for an additional 18-hour period in 10% FCS-DMEM. Cytotoxicity and cell death were always determined 18 h after SNP incubation (except for time course assay), using the assays described below.

### Inhibitors

The role of caspases was investigated by using a pancaspase inhibitor zVAD.fmk (0–100 μM). To assess the involvement of protein kinases, we used calphostin C (25 nM), SB 202190 (10 μM), and PD98059 (10 μM). SN-50 (0–50 μM) and PDTC (0–10 μM) were applied to investigate the role of nuclear translocation of NF-kB. Genistein (50 μM) was used as a tyrosine kinase inhibitor. To explore the effect of oxidative stress/free radicals in NO-mediated ligamentocyte apoptosis, scavengers for peroxynitrite (ONOO^-^) (uric acid, 0–2 mM), NO (carboxy-PTIO, 0–12.5 μM), and superoxide/hydrogen peroxide (O_2_^-^/H_2_O_2_) (taxifolin, 0–100 μM) were used. The COX-2 inhibitor NS-398 (0–100 μM) was used to examine the role of endogenously synthesized PGE_2_. Preliminary experiments were used to find inhibitor concentrations inducing maximal response in our study (data not shown). These different inhibitors were tested up to the maximal concentrations that were effective under the actual experimental conditions.

### Cytotoxicity assay

Cytotoxicity was determined as a function of the cellular conversion of the tetrazolium salt 3-(4,5-Dimethylthiazol-2-yl)-2,5-diphenyltetrazoliumbromid (MTT) into a DMSO soluble formazan product that was measured at 490 nm in a microplate reader (EL 800, BioTek, USA) as described before [24]. In all experiments using this assay, results were expressed as a reduction of cell viability (% of control cell cultures without any treatment) using the following formula with OD as optical density: Cell viability (%) = 100 × (Sample OD – Blank OD/Control OD – Blank OD).

### Apoptosis assay

Flow cytometry with fluorescein isothiocyanate (FITC)-conjugated annexin V and propidium iodide double staining was used to identify apoptotic cell death (FACS LRII, BD Biosciences). This assay allows detection of apoptotic cells by Annexin V binding, executed simultaneously with propidium iodide as a dye exclusion test [25]. Briefly, floating cells were collected and adherent cells were cautiously detached using trypsin/PBS. The cells were pooled, centrifuged at 1500 rpm and 4°C for 6 min. After decanting the supernatant, cells were resuspended in 800 μL ice-cold annexin V labeling buffer (10 mM HEPES, 140 mM NaCl, 2.5 mM CaCl_2_) for washing. After a second centrifugation, the pellets were resuspended in 100 μL annexin V labeling buffer added with FITC-conjugated annexin V to a final concentration of 0.1 μg/mL and incubated for 30 min at 4°C. Counterstaining with 5 μg propidium iodide/mL cell suspension was done 1 min before analyzing. Cell stained FITC-Annexin V positive and propidium iodide negative were detected as early apoptotic cells, demonstrating Annexin V binding and cytoplasmic membrane integrity. Late apoptotic cells and necrotic cells show Annexin V binding and propidium iodide uptake due to loss of cell membrane integrity and leakage of cellular constituents. Data files were generated for 5×10^4^ cells or more per sample using the FlowJo V7.1 Analysis Software (Treestar Inc., Ashland, OR, USA).

### Western immunoblot of COX-2 and cleaved caspase-3

For COX-2 and cleaved caspase-3 detection, canine cruciate ligamentocytes were lysed in RIPA buffer for 30 min. Twenty μg supernatant proteins were separated onto 12% Tris–HCl acrylamide gel and transferred onto nitrocellulose membrane. The membranes were blocked with 5% nonfat dry milk in TBS with 0.1% Tween-20, for 1 h at RT. The membranes were probed overnight at 4°C with a primary antibody (rabbit polyclonal anti murine COX-2, Cayman, 1:200, or rabbit monoclonal anti human cleaved caspase-3 (Asp175), Cell Signaling, 1:1000, both in 5% nonfat dry milk, TBS, 0.1% Tween-20). Thereafter blots were probed with the corresponding secondary antibody, goat-anti-rabbit Ig (HRP) (1:3000, DAKO, Switzerland). Immunoreactive signals were visualized by the ECL system (GE Healthcare, Switzerland) according to the manufacturer`s protocol.

### Prostaglandin E_2_ production

Prostaglandin E_2_ was determined in culture medium supernatants by the PGE_2_ EIA Kit following the company`s protocol (Cayman Chemical, Michigan, USA).

### Quantification of bcl-2 protein levels

Canine cruciate ligamentocytes were seeded at 10^6^ cells per well in a 6-well plate in 2 mL 10% FCS-DMEM and cultured until confluence. Cells were treated with different inhibitors and various concentrations of SNP. The bcl-2 protein levels of adherent cells were assayed using commercially available kits (human bcl-2 ELISA kit, Abnova, Germany) according to the manufacturer`s instructions. The level of bcl-2 was expressed in ng per milligram of total protein.

### Statistical analysis

All statistical analyses were accomplished using NCSS 2007 Statistical Software (http://www.ncss.com). Each data point represented the mean ± SD, of n different cell lines (i.e., donors) each performed in triplicates. Data normality was evaluated using the Shapiro-Wilk test. One-way analysis of variance (ANOVA) with post hoc Dunnett`s multiple comparisons was used for statistical comparisons to the control treatment. *P*-values < 0.05 were considered as significant.

## Results and discussion

Evidence documenting NO as a crucial mediator for canine CCL disease prompted our investigations to the underlying signaling of NO-induced cell death in cruciate ligamentocytes. Cruciate ligamentocytes are not only the sites of NO-production [9,22] but are also themselves targets for NO and undergo apoptosis upon exposure to high concentrations of NO [24]. Although CCL and CaCL have the same extrasynovial environment, apparently the same nutrition and blood supply, incidence of rupture differs between the ligaments. The rationale for using cells from the CCL as well as from the CaCL was that in a previous study we found different susceptibilities to apoptosis [24]. However, a comparison of the effect of pathway inhibitors on NO-induced cell death did not showed any significant differences between CCL and CaCL cells suggesting that the varying susceptibilities are not related to the signaling cascades which were analyzed below.

### Role of caspase-independent apoptosis and bcl-2 down-regulation in CCL and CaCL cells

**Figure 1:**
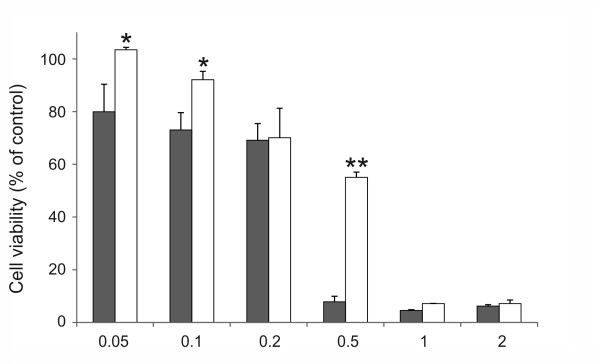
**Dose-dependent effect of SNP on cell viability in canine cruciate ligamentocytes.** Cells were cultured with indicated concentrations of SNP (mM) for 18 h. Cell viability of 
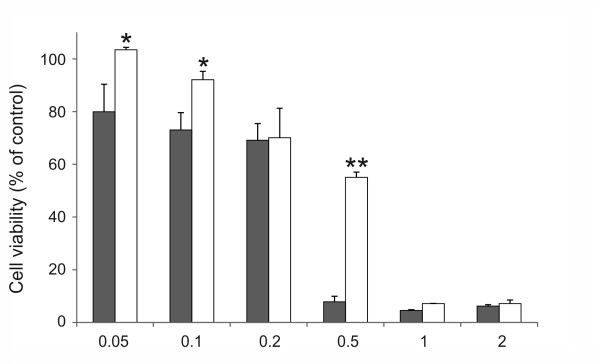
 CCL and □ CaCL cells was measured by MTT assay and calculated with the formula reported in the M&M section. Control cells were not treated with SNP. The graphs data represent the mean ± SD from at least three separate experiments of four different cell donors, each performed in triplicates. * *P* < 0.05, ** *P* < 0.01 CCL vs. CaCL at each indicated concentration.

**Figure 2:**
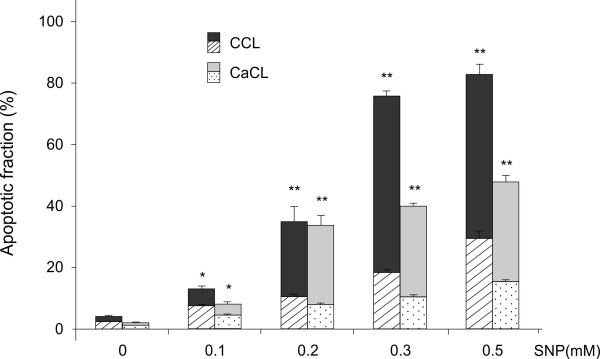
**Apoptotic fraction in canine cruciate ligament cells.** Canine CCL and CaCL cells were stimulated with indicated concentrations of SNP for 18 h. Apoptotic cells were measured by FITC-annexinV/propidium iodide double stained flow cytometry. The stacked bar graphs are divided into two categories: patterned graphs indicate the early apoptotic fractions detected as cells stained annexin V positive and propidium iodide negative, plain-colored graphs indicate the end stage apoptosis and death detected as cells stained annexin V and propidium iodide positive. The graphs data represent the mean ± SD from at least three separate experiments of four different cell donors, each performed in triplicates. * *P* < 0.05, ** *P* < 0.01 for combined (early and late) apoptotic fractions of CCL or CaCL treated with SNP at each indicated concentration vs. control without SNP treatment.

Cultures of canine cruciate ligamentocytes were stimulated with increasing concentrations of SNP, and cell viability was assessed by MTT assay and flow cytometry. A dose-dependent loss of cell viability was induced by SNP in CCL and CaCL cells (Figure 1). Using the double staining FITC-labeled Annexin V and propidium iodide flow cytometry, we could corroborate that CCL and CaCL cells died by apoptosis (Figure 2). Comparing CCL and CaCL cells, it became obvious that CaCL cells were less susceptible to NO-stimulated cell death. This effect was significant at 0.05 mM, 0.1 mM and 0.5 mM of SNP (Figure 1). In particular, at 0.5 mM of SNP, nearly all CCL cells were dead compared to CaCL cells (92% vs. 45%, *P* < 0.01). At concentration above 0.5 mM of SNP, viability of CaCL cells as well decreased to nearly the same amount of CCL cells. At high concentration of SNP, such as 0.5 mM for CCL cells and 1 mM for CaCL cells, the proportion of dead cells increases dramatically that we assume cell death might have changed its shape from apoptosis to necrosis. Although apoptosis and necrosis are commonly regarded as conceptually distinct modes of cell death, there is increasing evidence that the two classical types of demise can occur simultaneously in tissue or in cell cultures[26]. Thus, we used lower concentrations of SNP to investigate different pathways. The difference in susceptibility to apoptosis between the two cruciate ligaments was also demonstrated with other apoptosis inducer [24]. Measurement of the pro-survival bcl-2 protein showed that the level decreased in association with SNP treatment in a dose-dependent way (Table 1). In healthy cells, basal levels of anti-apoptotic proteins like bcl-2 protein promotes cell survival by inhibition adapters needed for activation of caspases that dismantle the cell [27]. The effect of SNP on bcl-2 levels in cruciate ligamentocytes is consistent with the effect described in human OA chondrocytes [19].

**Table 1:** Dose-dependent effect of SNP with/without inhibitors on bcl-2 protein level in canine CCL and CaCL cells

	bcl-2 [ng/mg protein]	
	**CCL cells (n = 6)**	**CaCL cells (n = 6)**
Control	6.6 ± 0.3	7.0 ± 0.4
SNP (0.3 mM)	6.5 ± 0.1	5.1 ± 0.3*
SNP (0.5 mM)	5.5 ± 0.3*	4.9 ± 0.2*
SNP (1 mM)	2.8 ± 0.2**	3.9 ± 0.1**
SNP (0.5 mM) + Genistein (50 μM)	8.8 ± 0.2††	10.9 ± 0.4††
SNP (0.5 mM) + Uric acid (0.5 mM)	7.8 ± 0.1††	7.9 ± 0.1††
SNP (0.5 mM) + Taxifolin (50 μM)	7.6 ± 0.3†	6.7 ± 0.4†
SNP (0.5 mM) + PTIO (5 μM)	5.8 ± 0.2	5.1 ± 0.2

Data represent the means and standard deviations from two separate experiments, n refers to the number of donors, and each performed in triplicates. Bcl-2 protein was measured by enzyme immunoassay. *, *P* < 0.05, **, *P* < 0.01 vs. control; †, *P* < 0.05, ††, *P* < 0.01 vs. SNP (0.5 mM); Dunnett`s multiple comparison test.

**Figure 3:**
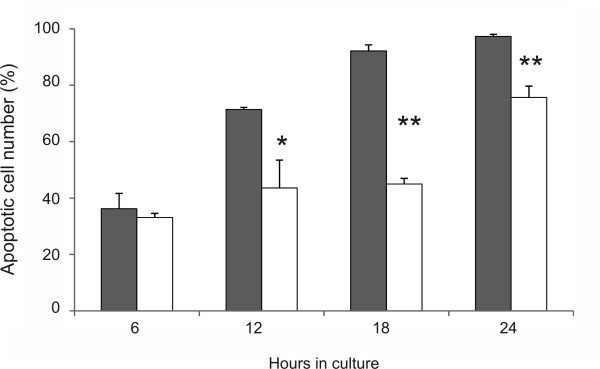
**Time-dependent effect of SNP on canine cruciate ligamentocytes.** The apoptotic cell number in canine CCL and □ CaCL cells was measured by flow cytometry. Cells were incubated in medium with 0.5 mM SNP for 6, 12, 18 and 24 h. Values correspond to the mean ± SD from at least three separate experiments of four different cell donors, each performed in triplicates. *, *P* < 0.05, **, *P* > 0.01 CCL vs. CaCL at each indicated time.

Time course experiments demonstrated that at 0.5 mM, SNP induced an earlier reduction of cell viability in CCL cells than in CaCL cells (Figure 3). This time-dependent effect was significantly apparent at 12 to 24 h. This dose- and time-dependent manner of apoptosis induction is closely in agreement with different studies using SNP-generated NO to stimulate various human and rabbit cell types originating from stifle tissue [19-21]. In those studies, cell death was clearly linked to the activation of the caspase cascade, as inhibitors of caspase-3 or caspase-9 prevented the cells from apoptosis. In canine cruciate ligamentocytes, induction of cell loss by SNP was only marginally prevented when the cells were prestimulated for 2 h with 100 μM of the pancaspase inhibitor zVAD.fmk (Table 2). Same results were achieved in a recent study with other NO-donors such as DETA or SNAP [24]. We then tested whether caspase-3 was activated by SNP. While caspase-3 processing was induced by staurosporine which served as positive control, no evidence for caspase-3 cleavage was found by immunoblotting in SNP-treated cruciate ligamentocytes in the concentration range in which apoptosis was measured by flow cytometry (data not shown). These observations suggest a participation of a caspase-independent cell death pathway (CICD), which has been noticed in the presence of the broad-spectrum caspase inhibitor [28]. Evidence suggests the existence of CICD that is mediated by apoptosis-inducing factor (AIF), a mitochondrial flavoprotein, which translocates to the nucleus and induces chromatin condensation and DNA fragmentation [29]. Typically, mitochondrial functions decline during CICD, although this is generally a slower process than seen in apoptosis [30].

**Table 2:** Cell viability of canine cruciate ligamentocytes in response to different inhibitors and SNP treatment

Inhibitor pretreatment		CCL cell viability (n = 8)		CaCL cell viability (n = 8)	
		SNP treatment		SNP treatment	
		-	+	-	+
zVAD.fmk	0	100 ± 6.0	75.5 ± 3.1	100 ± 5.9	73.6 ± 2.4
	100 μM	92.5 ± 5.3	84.5 ± 4.3	92.2 ± 6.3	88.2 ± 3.6*
CalC	0	100 ± 11.3	79.9 ± 5.3	100 ± 5.9	78.2 ± 7.2
	25 nM	93.5 ± 6.9	73.4 ± 3.3	100 ± 6.0	68.7 ± 7.0
PD98059	0	100 ± 5.6	75.8 ±6.4	100 ± 4.1	74.7 ± 9. 3
	10 μM	81.2 ± 7.2*	61.8 ± 3.1*	78.3 ± 7.2*	52.2 ± 9.2*
SB202190	0	100 ±11.3	73.4 ± 5.4	100 ± 6.0	74.2 ± 7.7
	10 μM	74.2 ±2.1*	65.9 ± 4.5	75.2 ± 5.8*	79.8 ± 2.4
Genistein	0	100 ± 6.0	69.6 ± 6.1	100 ± 5.8	72.3 ± 5.7
	50 μM	101 ± 6.4	97.4 ±9.3**	106.1 ±6.3	101.9 ± 2.5**
NS-398	0	100 ± 5.4	64.9 ± 3.6	100 ± 5.4	63.2 ± 4.1
	50 μM	89.5 ± 17.7	78.1 ± 3.9*	88.5 ± 4.7*	88.8 ± 2.5**
	100 μM	74.7 ± 14.4	75.3 ± 4.2*	81.8 ± 12.1	92.4 ± 2.7**
SN-50	0	100 ± 8.3	74.6 ± 3.8	100 ± 8.4	81.2 ± 2.4
	50 μM	97.3 ± 8.6	64.4 ± 12.6	101.4 ± 4.5	67.3 ± 2.7*
PDTC	0	100 ± 8.3	74.6 ± 3.8	100 ± 8.4	81.2 ± 2.4
	10 μM	42.7 ± 7.7**	5.5 ± 1.9**	43.8 ± 2.3**	2.1 ± 1.7**
Uric acid	0	100 ± 9.2	73.5 ± 5.4	100 ± 5.6	78.2 ± 2.4
	0.5 mM	103.6 ± 9.5	102.7 ± 12.1*	101.9 ± 6.2	94.6 ± 5.9*
PTIO	0	100 ± 6.2	64.9 ± 2.6	100 ± 9.3	61.2 ± 4.6
	5 μM	87.9 ± 7.3	80.9 ± 1.5*	96.2 ± 9.6	73.8 ± 3.2*
Taxifolin	0	100 ± 3.7	76.2 ± 4.2	100 ± 5.6	74.3 ± 7.7
	100 μM	91.6 ± 11.5	84.8 ± 10.6	101.3 ± 4.1	97.3 ± 6.8*

Values correspond to the mean ± SD, calculated by using the formula from M&M section of three separate experiments of n different cell donors, each performed in triplicates. Cruciate ligamentocytes were preincubated with the indicated concentrations of the inhibitors for 2 h. SNP or none SNP were then added to the cultures and allowed to incubate for an additional 18-h period. SNP concentrations used were 0.2 to 0.25 mM. Cell viability was assayed by MTT assay. *P-*values indicate difference within the same type of cell and SNP treatment versus absent inhibitor: *, *P* < 0.05; **, *P* < 0.01.

### Role of mitogen-activated protein kinase, protein kinase C, tyrosine kinase, and NF-kB inhibitors on SNP-induced cruciate ligamentocyte death

MAP kinase is a family of enzymes that play an important role in converting extracellular signals to intracellular messengers that regulate several cellular phenomena, including apoptotic cell death or survival [31]. The issue of whether MAP kinase activation determines cell survival or death remains controversial. Several studies indicate that Mitogen activated protein kinase subtypes ERK1/2 are activated in response to mitogen or growth factor stimulation and that its activation is coupled with cell survival [32,33]. The subtype p38 kinase is stimulated during cellular stress conditions and its activation is associated with apoptosis [20,33]. Previous studies in humans and in animals like dogs or rabbits demonstrate that NO represent a severe stress factor for normal or OA chondrocytes [19,20,34]. They demonstrated that exogenous or endogenous NO induce the activation of MAP kinase p38 and ERK1/2 because interruption of the kinase signaling by using ERK1/2 inhibitor (PD98059) and MAP kinase p38 inhibitor (SB202190) significantly influenced apoptosis in two different directions. To elucidate the role of potential upstream signaling molecules in canine cruciate ligamentocytes, we used the same cell-permeable inhibitors SB202190 and PD98059. Inhibition of ERK1/2 by PD98059 (10 μM) caused death equally in both canine cruciate ligamentocytes which was significantly pronounced in case of a coincubation with SNP (Table 2). This indicates that ERK1/2 function as anti-apoptotic signals in canine cruciate ligament cells which is in accord with that reported for rabbit chondrocytes [20], but differ from the reaction in human and canine OA chondrocytes which in contrast could be protected against apoptosis by using PD98059 [19,34]. Inhibition of p38 kinase with SB202190 significantly block NO-induced apoptosis in several cell types like human [19] and canine [34] OA chondrocytes as well as normal rabbit chondrocytes [20]. However, in CCL and CaCL cells, the p38 inhibitor did not reduce apoptosis excluding p38 kinase as a pro-apoptotic signaling cascade upon NO. Blocking another signaling cascade by calphostin C, cell loss through SNP was not significantly inhibited in both canine cruciate ligamentocytes, suggesting that SNP-induced cell death was not mediated by protein kinase C. In contrast, this pathway is activated in human OA chondrocytes and synoviocytes [19,21]. To improve functionality of calphostin C, we performed the same experiments on human OA chondrocytes which served as positive controls and could verify the protective effect of these inhibitors as reported [19].

In an attempt to elucidate the potential role of NF-kB regulated apoptosis in canine cruciate ligamentocytes, we used two inhibitors. SN-50 inhibits nuclear translocation of the activated NF-kB complex, whereas PDTC prevents the activation of NF-kB. The activation of NF-kB can have both preventative and causative roles in the induction of apoptosis [35]. In our cells, PDTC (10 μM) alone showed a significant reduction in viability and reduced the number of viable cells below 5% after SNP coincubation in CCL and CaCL cells. The effect was enhanced by addition of SNP. An apoptosis-enhancing capability was also seen when cells were treated with SN-50 (50 μM) following coincubation with SNP (Table 2) but the effect was less pronounced. This apoptosis–enhancing capability of NF-kB inhibitors is consistent with studies on human OA chondrocytes [19].

Nitric oxide donors have the capacity to cause phosphorylation of various targets, including the tyrosine kinase (TK). The TK family plays a key role in regulation of cell proliferation, differentiation, metabolism, as well as survival [36]. They catalyze the transfer of γ-phoshoryl groups from ATP to tyrosine hydroxyls of proteins. The phosphorylation of tyrosine residues modulates enzymatic activity and creates binding sites for recruitment of downstream signaling proteins. The TK inhibitor genistein is involved in different cell processes such as inhibition of tumor cell proliferation, activation of tumor cell differentiation as well as blocking oxidative DNA damage in vitro [37,38]. Previous studies showed that blocking TK with genistein was very effective in preventing apoptosis in human OA chondrocytes and synoviocytes after SNP exposure [19,21]. Likewise, in canine cruciate ligamentocytes, genistein markedly and dose-dependently preserved cell viability in the presence of SNP (Table 2). The protective effect was not statistically different between CCL and CaCL but was the highest of all used specific pharmacological inhibitors in our study. Even strong cytotoxic effects of high SNP concentrations (0.5 mM) were effectively degraded (from about 80% apoptosis to less than 20% in CCL and 13% in CaCL cells). Furthermore, addition of genistein to CCL and CaCL cells exposed to SNP resulted in a 1.6-fold and 2.2-fold increase in the bcl-2 protein level, relative to cruciate ligamentocytes exposed to SNP in the absence of the inhibitor (Table 1). Furthermore, the protection was associated with a complete inhibiton of PGE_2_ secretion. This implies that TK is an important transducing pathway in regulating cellular susceptibility to NO in canine cruciate ligamentocytes, a finding similar to that reported in human OA chondrocytes and synoviocytes [19,21].

### Role of ROS in SNP-induced cruciate ligament cell death

In a previous study application of iNOS inhibitors was not able to reduce apoptosis in CCLs in vivo [39] suggesting NO does not feature the sole responsibility for CCL cell apoptosis. Several possible systems have been considered to explain the exact mechanisms of NO-mediated cytotoxicity. Because of the potential of NO to react with free radicals, we investigated whether SNP caused toxicity directly, due to NO, or indirectly, due to ROS formation. In the present study, ROS were found to be a major activation pathway in SNP-induced cruciate ligamentocyte cytotoxicity. We demonstrated that blocking generation of ROS significantly attenuate apoptosis. Taxifolin, a scavenger of O_2_^-^ and H_2_O_2_, and the NO-scavenger carboxy-PTIO significantly reduced death in cranial as well as in caudal cruciate ligamentocytes (Table 2). The most pronounced effect was reached by using the ONOO^-^ scavenger uric acid. Uric acid protected cruciate ligamentocytes against the toxic effect of NO in a dose-dependent manner and significantly increased their viability. In human OA, the uncontrolled production of free radicals is considered an important factor in the pathogenesis of OA [40,41]. Del Carlo and Loeser demonstrated that chondrocyte cell death from NO occurred only under conditions where other ROS were concurrently generated [42]. The NO-scavenger PTIO showed a protective effect on SNP-induced cell death in human OA and normal chondrocytes, whereas the ROS scavenger N-acetyl-L-cysteine profoundly blocked DNA degradation in normal human chondrocytes [21,43]. On the level of bcl-2, ROS inhibitors like uric acid and taxifolin prevented a downregulation of bcl-2 protein in SNP-treated cruciate ligamentocytes (Table 1).

### Role of COX-2 and PGE_2_ in SNP-mediated cruciate ligament cell death

In vivo and in vitro studies observed that iNOS and COX-2 are induced in a number of inflammatory models including human OA [19,21] and canine OA [34]. Based on the potential crosstalk between the two systems and the clinical usage of COX inhibitors in orthopedic diseases in dogs, we were interested whether such a regulation exists in stress-induced cruciate ligamentocytes as well. The COX-2 inhibitor NS-398 (0–100 μM) dose-dependently and significantly attenuated the cytotoxic effect of NO in both cruciate ligamentocytes (Table 2), but it was less clearly than reported in human OA chondrocytes or synoviocytes where NS-398 totally abolished the effect of NO [19,21]. In experimental OA in dogs, specific inhibition of COX-2 by NS-398 significantly reduced the level of chondrocyte death [34].

Nitric oxide has the ability to activate COX-1 and COX-2 enzymes and hence PG production [44]. Under normal conditions small amounts of PG are released by constitutively expressed COX-1 and exert beneficial cytoprotective effects, whereas high PG concentrations were released by COX-2 at site of inflammation [45]. In canine cruciate ligament cells, measurements of PGE_2_ concentrations showed that endogenous PGE_2_ release was increased dose-dependently by SNP treatment (Table 3). The amount of PGE_2_ in cruciate ligamentocytes was significantly lower compared to the amount in human OA chondrocytes measured in parallel (Table 3) but no differences were found between CCL and CaCL. As a limitation of the study, we were not able to attribute the increase of PGE_2_ to COX-1 or COX-2 activation because we only performed COX-2 immunoblotting. COX-2 expression was not detected in SNP-treated canine cruciate ligament cells, but the quantity of COX-2 protein was possibly below the detection limit (data not shown). In contrast, in sections of canine OA cartilage COX-2 could be detected in situ by immunohistochemistry [34]. These data suggest that the COX-2 system exhibits a less important regulator of NO-induced cell death in canine cruciate ligament cells than for example in canine OA chondrocytes [34]. The low concentrations of PGE_2_ and the undetectable COX-2 expression could be an indication for a lower metabolic activity of cruciate ligamentocytes in inflammatory processes. Investigation of PGE_2_ in cruciate ligamentocytes from dogs with cruciate disease would be helpful to prove if a low PG activity is phenotype (normal or OA cell) independent and hence specific in canine cruciate ligamentocytes.

**Table 3:** **Dose-dependent effect of SNP and inhibitors on PGE**_**2**_**production in canine CCL and CaCL cells**

	**PGE_2_ [pg/mL/2 × 10^6^ cells]**		
**Treatment**	**CCL cells**	**CaCL cells**	**Human chondrocytes**
Control	35.4 ± 0.1	29.0 ± 0.6	620 ± 10
SNP (0.2 mM)	90.1 ± 1.3*	81.8 ± 2.2*	995 ± 30*
SNP (0.4 mM)	134.2 ± 5.4*	91.6 ± 14.5*	
SNP (0.5 mM)	247.2 ± 16.9*	108.6 ± 10.6*	1636 ± 22*
SNP (1.0 mM)	239.4 ± 12.6*	179.8 ± 8.2*	12790 ± 870*
SNP (0.2 mM) + Genistein (50 μM)	39.5 ± 3.4†	43.1 ± 2.3†	
SNP (0.2 mM) + NS-398 (50 μM)	46.2 ± 2.1†	46.3 ± 3.8†	

Data are the mean ± SD from two separate experiments each performed in triplicates. PGE_2_ release in pg of 2 × 10^6^ cells into 1 mL medium was measured by enzyme immunoassay. Human chondrocytes served as positive controls. *, *P* < 0.01 vs. control; †, *P* < 0.01 vs. SNP (0.2 mM); Dunnett`s multiple comparison test.

A possible limitation of our data is the fact that we used cells from healthy joint tissue and compared the results to published studies using healthy and OA tissue. However, these studies show that the same principle pathways exist in OA and healthy cells independent on their phenotype but might have different degree of activation [19,20,32,34].

## Conclusions

Canine cranial cruciate ligament cells clearly showed a higher susceptibility to NO-induced apoptosis. Albeit we could not find any significant differences between CCL and CaCL cells in the tested pathways, suggesting that the different susceptibility to apoptosis was not related to these pathways. A comparison of canine cruciate ligament cells with other cells from articular tissue demonstrates that cruciate ligamentocytes have a specific signaling on the stressor NO. Nitric oxide induced ligament cell death seemed to be mediated multi-plain whereupon TK and ROS play a major role. New efforts to prevent the development and progression of OA may include strategies and interventions aimed at reducing oxidative damage. Further studies are necessary to clarify if the inhibition of apoptosis in cruciate ligamentocytes induced by NO or other stressors would be effective in preservation ligament homeostasis. Until now the standard of care for CCL disease is surgical. With this study in perspective, we open the discussion to the use of disease-modifying therapy as a less invasive procedure offering a potential solution for CCL regeneration.

## Competing interests

The authors declare that they have no competing interests that could inappropriately influence or bias the content of the paper.

## Author’s contribution

SF designed the study, carried out the experiments, interpreted the data, and drafted the manuscript. AZ participated in its design and helped in interpretation of data. DS conceived of the study and coordinated and helped to draft the manuscript. All authors read and approved the final manuscript.
